# Monitoring type 2 diabetes from volatile faecal metabolome in Cushing’s syndrome and single *Afmid* mouse models via a longitudinal study

**DOI:** 10.1038/s41598-019-55339-9

**Published:** 2019-12-11

**Authors:** Célia Lourenço, Darren Kelly, Jack Cantillon, Michael Cauchi, Marianne A. Yon, Liz Bentley, Roger D. Cox, Claire Turner

**Affiliations:** 1 School of Life, Health & Chemical Sciences, Faculty of Science, Technology, Engineering and Mathematics, The Open University, Walton Hall, Milton Keynes, MK7 6AA UK; 2 Now at: School of Water, Energy and Environment, Cranfield University, Cranfield, Bedfordshire MK43 0AL UK; 3 Department of Mathematics & Statistics, University of Limerick, Limerick, V94 T9PX Ireland; 4 Mammalian Genetics Unit, MRC Harwell Institute, Harwell Campus, Oxfordshire, OX11 0RD UK; 5 Present Address: College of Health & Life Sciences, Brunel University London, Kingston Lane, Uxbridge, Middlesex UB8 3PH UK

**Keywords:** Metabolomics, Type 2 diabetes, Prognostic markers

## Abstract

The analysis of volatile organic compounds (VOCs) as a non-invasive method for disease monitoring, such as type 2 diabetes (T2D) has shown potential over the years although not yet set in clinical practice. Longitudinal studies to date are limited and the understanding of the underlying VOC emission over the age is poorly understood. This study investigated longitudinal changes in VOCs present in faecal headspace in two mouse models of T2D – Cushing’s syndrome and single *Afmid* knockout mice. Longitudinal changes in bodyweight, blood glucose levels and plasma insulin concentration were also reported. Faecal headspace analysis was carried out using selected ion flow tube mass spectrometry (SIFT-MS) and thermal desorption coupled to gas chromatography-mass spectrometry (TD-GC-MS). Multivariate data analysis of the VOC profile showed differences mainly in acetic acid and butyric acid able to discriminate the groups *Afmid* and Cushing’s mice. Moreover, multivariate data analysis revealed statistically significant differences in VOCs between Cushing’s mice/wild-type (WT) littermates, mainly short-chain fatty acids (SCFAs), ketones, and alcohols, and longitudinal differences mainly attributed to methanol, ethanol and acetone. *Afmid* mice did not present statistically significant differences in their volatile faecal metabolome when compared to their respective WT littermates. The findings suggested that mice developed a diabetic phenotype and that the altered VOC profile may imply a related change in gut microbiota, particularly in Cushing’s mice. Furthermore, this study provided major evidence of age-related changes on the volatile profile of diabetic mice.

## Introduction

Volatile organic compounds (VOCs) are produced as a consequence of endogenous metabolic activities and these can potentially be used as an alternative non-invasive approach to assist in the diagnosis or in monitoring the progress of disease. The biochemical origin of VOCs in exhaled breath, skin, urine and faecal headspace in rodent models is far from being well understood, and published literature is scarce. Breath analysis in mice is complex, the sampling is challenging, and the detection of VOC at trace concentration with enough sensitivity is problematic^1–6^.

Changes in VOCs emitted from stools can provide insight into the metabolic functions of the gut microbiota. Gut microbiota are thought to influence glucose and energy metabolism through the production of short-chain fatty acids (SCFAs). These volatile SCFAs include butyric acid, acetic acid, propionic acid, formic acid, isobutyric acid, valeric acid, isovaleric acid, and caproic acids, although acetate, propionate and butyrate make up 90 to 95% of SCFAs within the gut^7,8^. These fermentation products (SCFAs) are mainly generated from gut bacterial degradation of undigested dietary carbohydrates, along with the production of CO_2_, H_2_, and CH_4_^9^. In addition, amino acids (valine, leucine and isoleucine) can be further degraded into isobutyrate, isovalerate and 2-methylbutyrate^10^. The composition of the gut microbiota is subject to genetic and environmental factors and notably it changes with age^11^. Longitudinal studies are scarce in literature and these yield numerous advantages compared to a single-cohort, such as assessing intra-individual variability, and in providing information about the onset and progress of disease.

Type 2 diabetes is a metabolic disease which has been attributed to changes in diet, poor physical activity, life-style and genetic predisposition, while more recently there is increasing evidence of a link between metabolic diseases and gut microbiota composition, with microbial metabolites having a major influence on host physiology. Alterations to the gut microbiome have been linked to obesity, insulin resistance and type 2 diabetes^12–16^. However, whether gut microbiota plays an underlying role in the biological mechanisms that leads to the onset of metabolic diseases is unknown, and the understanding of such metabolic pathways in humans is limited. There is evidence of changes in the composition of faecal microbiota of type-2 diabetic patients when compared to non-diabetic subjects, where the relative abundance of bacterial species *Firmicutes* was significantly lower, while the proportion of *Bacteroidetes* and *Proteobacteria* was somewhat higher in diabetics compared to non-diabetic controls^17^.

Initial studies using mouse models are useful to provide insight about the disease at controlled conditions. The mouse model of Cushing’s syndrome used in this study is a *N*-ethyl-*N*-nitrosourea (ENU) chemically induced point mutation in the *Crh* promoter (the point mutation is inherited) and is a model with many of the features of type 2 diabetes^18^. These Cushing´s mice develop excessive circulating glucocorticoid concentrations in the body. Glucocorticoid is involved in the metabolism of carbohydrates, proteins and fats. As a consequence, mice are prone to develop obesity, hyperglycaemia, and insulin resistance, along with hair loss, thin skin and low bone mineral density^6,18,19^.

The enzyme arylformamidase (*Afmid*), also known as kynurenine formamidase participates in tryptophan metabolism, which in turn regulates many functions including pancreatic secretion, among others^20^. The *Afmid* knockout mice have been used to further investigate possible links between abnormal tryptophan metabolism and diabetes. The phenotype of these mice include a defect in glucose stimulated insulin secretion and reduced islet mass with age. These mice show impaired glucose tolerance, although their insulin sensitivity is unchanged when compared to wild-type (WT) animals^6,21^.

Metabolomics enables the exploration of disease-related metabolites, the so-called unique *fingerprint* of VOCs, through the aid of highly sensitive detection methods. Selected ion flow tube mass spectrometry (SIFT-MS) and thermal desorption coupled to gas chromatography-mass spectrometry (TD-GC-MS) have been widely used for untargeted analysis, and when combined these techniques provide a powerful approach for the detection and identification of metabolites in a biological sample^22–26^. In the present study we investigated the longitudinal changes in volatile faecal metabolites in mouse models of diabetes acquired either by SIFT-MS and TD-GC-MS. We employed multivariate statistics to demonstrate that differences in VOCs can accurately classify samples based upon phenotype. This is a novel piece of work that aimed to study the progress of diabetes in mice and to understand the gut metabolomics through the analysis of the volatile faecal profile and through the aid of highly sensitive analytical techniques.

## Results and Discussion

### The onset of obesity and dysregulation of plasma insulin and blood glucose

**Figure 1:**
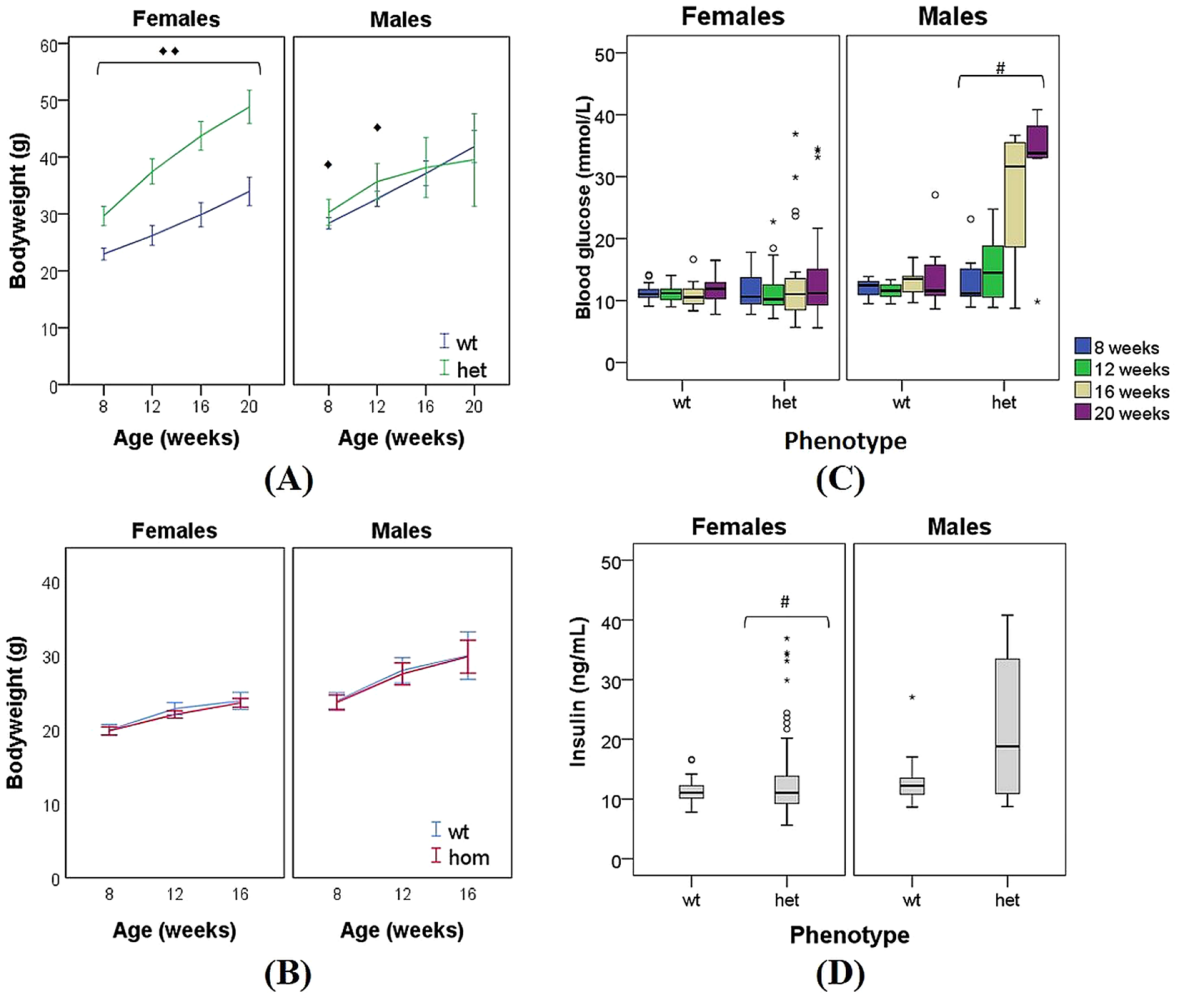
Changes in bodyweight (expressed in g) over the age of female and male (**A**) Cushing’s (*het*) mice and (**B**) Afmid (*hom*) mice compared with their respective WT littermates. Highlighted in green (*het*); highlighted in red (*hom*); highlighted in blue (WT) **(C)** Blood glucose concentrations expressed in mmol L^−1^ of Cushing’s mice (*het*) compared with WT littermates, age- and sex-matched groups **(D)** plasma insulin concentrations expressed in ng mL^−1^ of Cushing’s mice (*het*) compared with WT littermates, age- and sex-matched groups. P values were calculated between age- and sex-matched groups using student’s t-test and one-way repeated measures ANOVA with Bonferroni post-hoc test (^◆^p < 0.05, ^◆◆^p < 0.001); Mann Whitney’s test (^#^p < 0.05). Outliers are represented with a circle (◦) and extreme points are indicated with an asterisk (*). Error bars represent SEM (standard error of mean).

A student’s t-test was used to compare the mean score of bodyweights (normally distributed) between Cushing’s (*het*) mice and WT littermates and between sex-matched groups. The mutation had statistically significant effects on bodyweight (Fig. 1A) in agreement with published literature^18^. Female mutant (*het*) mice were significantly heavier (p < 0.001) than the WT littermates on a B6-C3PDE background. While Cushing’s (*het*) male mice were significantly heavier than the respective WT littermates from 8 to 12 weeks of age. From 12 weeks onwards, Cushing’s (*het*) male mice developed increasing and detrimental levels of blood glucose (Fig. 1C) which in turn triggers the decrease in bodyweight of the animals. A one-way repeated measure ANOVA with a Bonferroni post-hoc test was conducted to compare the animal’s bodyweight throughout the age. The results indicated that either for females or males the bodyweight of the animals significantly changed over the four months of study (p < 0.001).

Disease was tracked for *Afmid* (*hom*) mice and their respective WT littermates across three time points, 8, 12 and 16 weeks of age (Fig. 1B). A Mann Whitney U test was used to compare the mean score of bodyweights between *Afmid* mice and WT littermates and between sex-matched groups. The non-parametric Friedman test (repeated measures) combined with individual post-hoc tests, i.e. Wilcoxon Signed Rank Test using a Bonferroni adjusted p value (p < 0.001), was used to compare the animal’s bodyweight over the age and to establish the statistically significant difference among the three time points. The results indicated that the bodyweight of the animals significantly changed over the age (p < 0.001), although there is no significant difference in the bodyweight between *Afmid* (*hom*) and WT littermates (Fig. 1B). *Afmid* (*hom*) male mice are significantly heavier (p < 0.05) than females over the three time points. Diet is a major factor that drives gut microbiota composition and the microbial generated metabolites^27,28^. In this study, all the animals (*het*, *hom* and WT) were under the same diet although we do not know whether diabetic animals ate the same amount as their respective WT littermates.

In Cushing’s mice, the mutation had statistically significant (p < 0.05) effects on blood glucose. The data indicated that male and female mutant (*het*) mice had hyperglycaemia (blood glucose > 11.1 mmol L^−1^) by the age of 16 weeks (Fig. 1C). In addition, the Cushing’s male mice developed significant increased concentrations of blood glucose from 16 weeks of age compared to WT littermates. Blood glucose sampling in mice induces stress in the animals and blood glucose variability might arise from that fact. In addition, some mice are more susceptible to obesity and diabetes than others, and insulin resistance manifested at different time points. Importantly, the estrous cycle in females significantly adds some variability into the data.

Overall mutant (*het*) animals had statistically significant elevated plasma insulin concentrations (median = 12, n = 30) after 20 weeks of age compared to WT littermates (median = 2.3, n = 35, p < 0.05) (Fig. 1D). In particular, Cushing’s (*het*) female mice developed statistically significant (p < 0.05) elevated plasma insulin concentrations compared to female WT littermates (Fig. 1D). Insulin secretion is triggered by elevated glucose concentration in the blood, supporting the hypothesis that Cushing’s mice develop a diabetic phenotype. Insulin differences between Cushing’s (*het*) male mice and male WT do not appear to be statistically significant.

*Afmid* (*hom*) male mice developed significant increased concentrations of blood glucose (p < 0.05) at the three time points compared to the respective WT littermates, although the increase of blood glucose levels with age is not statistically significant. People with impaired glucose tolerance (IGT) have plasma levels ≥ 7.8 and < 11.1 mmol L^−1^ glucose^29^. *Afmid* (*hom*) mice show impaired glucose tolerance and Mann Whitney U test revealed no significant difference in plasma insulin concentration of *Afmid* (*hom*) mice and WT littermates which is in agreement with earlier reported^21,30^.

### Multivariate statistical analysis on VOC emissions from stools

#### Discrimination of groups according to the phenotype – SIFT-MS and GC-MS data

**Figure 2:**
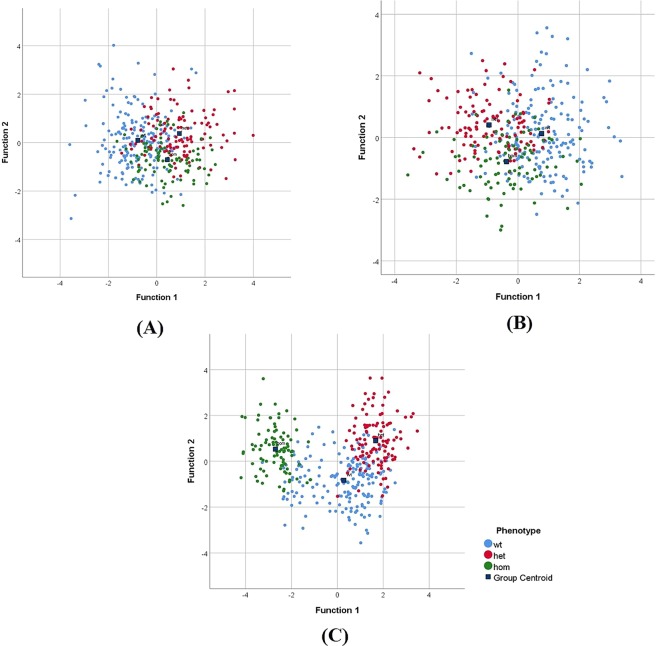
Canonical discriminant functions of the first two discriminant function values, revealing classification of groups (*wt*, *het*, *hom*) according to phenotype. Number of independent observations and number of variables is given in Supplementary Information (Table S1). Blue markers were assigned to WT littermates; red markers: Cushing’s (*het*) diabetic animals; green markers: *Afmid* (*hom*) animals. Data acquired using SIFT-MS (**A**) H_3_O^+^, (**B**) NO^+^ and (**C**) O_2_^+^.

The volatile faecal metabolome of the animals (wild-type *(wt)*, Cushing’s *(het)* and *Afmid (hom)*) was acquired using selected ion flow tube mass spectrometry (SIFT-MS) in the form of *m/z* versus counts per second and across the three precursor ions H_3_O^+^, NO^+^, O_2_^+^. Follow-up using the canonical discriminant functions (Fig. 2) was used to determine the group membership by finding linear combinations of the variables (*m/z*) that maximize the differences between the groups (*wt, het*, *hom*).

Overall, group separation was observed between Cushing’s *(het)* and *Afmid (hom) mice* and notably pronounced on the volatile profile acquired using O_2_^+^ precursor ion. Discrimination of groups *(hom, het)* and the WT animals was not entirely clear once analysing the data sets acquired with H_3_O^+^ and NO^+^ precursor ions, and therefore these findings were further investigated using partial least squares-discriminant analysis (PLS-DA) in conjunction with bootstrapping which provides a more robust approach.

The cross-validated classification using linear discriminant analysis (LDA) and PLS-DA results presented in Table 1 indicates the inter-group classification accuracy (*het*/WT) and the corresponding test sensitivity and specificity determined between Cushing’s (*het*) mice and WT littermates. *Afmid* (*hom*) knockout mice did not present statistically significant differences in their volatile faecal metabolome once compared to their WT littermates, on analysis with PLS-DA. The best classification accuracy results (*het/WT*) are highlighted in bold (Table 1). LDA was only performed on SIFT-MS data, whereas PLS-DA was performed on both data sets (SIFT-MS and GC-MS). The cross-validated classification showed that overall 89.2% were correctly classified using H_3_O^+^ data set at 8 weeks of age and 86.2% were correctly classified at 20 weeks. Sensitivities and specificities are given respectively. The statistical analysis performed demonstrated that 83.1% were correctly classified using NO^+^ data set at 8 weeks of age and 80.9% at 12 weeks. The best classification results are given for using O_2_^+^ data set at 12 and 16 weeks of age, where the cross-validated classification showed an overall 85.1% and 92.2% correct classification respectively.

**Table 1:** Cross-validated linear discriminant analysis (LDA) and partial least squares-discriminant analysis (PLS-DA) between Cushing’s (*het*) mice and WT littermates. Accuracy (%) and the corresponding test specificity and sensitivity given for faecal headspace of Cushing’s mice.

Statistical method	SIFT-MS (Ion)	Age (weeks)	Accuracy (%)		Specificity (%)		Sensitivity (%)	
			SIFT-MS	GC-MS	SIFT-MS	GC-MS	SIFT-MS	GC-MS
LDA	H_3_O^+^	**8**	**89.2**	N/A	91	N/A	87	N/A
		**12**	**81.5**		86		77	
		16	75.4		83		67	
		**20**	**86.2**		89		83	
		all	71.9		76		68	
LDA	NO^+^	**8**	**83.1**	N/A	89	N/A	77	N/A
		**12**	**80.9**		83		79	
		16	73.8		94		50	
		20	70.8		71		70	
		all	75.2		84		65	
LDA	O_2_^+^	8	81.5	N/A	89	N/A	73	N/A
		**12**	**85.1**		87		83	
		**16**	**92.2**		94		90	
		20	72.3		86		57	
		all	73.0		78		68	
PLS-DA	H_3_O^+^/NO^+^/O_2_^+^	8	67.9	68.0	82.6	78.4	50.0	55.4
		**12**	**70.8**	**64.1**	67.0	63.3	74.6	65.1
		**16**	**64.1**	**71.7**	68.0	76.3	59.9	66.0
		20	66.2	71.5	76.1	76.7	56.2	65.1

Data acquired using SIFT-MS and GC-MS coupled to thermal desorption (TD). H_3_O^+^/NO^+^/O_2_^+^ denotes the combination of the individual mass spectra pertaining to the three precursor ions into one dataset. The best classification results are highlighted in bold. N/A stands for non-applicable.

**Figure 3:**
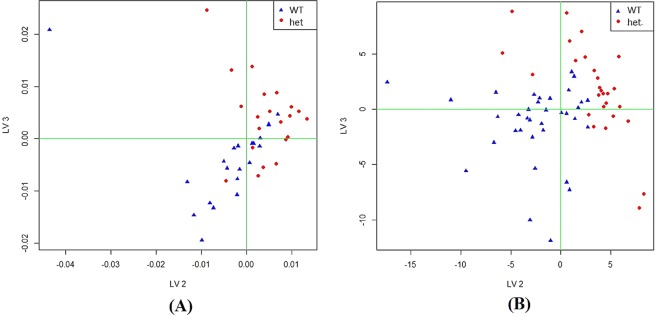
PLS-DA score plots discriminating between WT littermates and Cushing’s (*het*) mice at (**A**) 12 weeks of age including Feature Selection (FS), data acquired using SIFT-MS; (**B**) 16 weeks of age, data acquired by TD-GC-MS. Highlighted in blue is shown the WT littermates and Cushing’s (*het*) diabetic animals are assigned in red. Number of independent observations and number of variables is given in Supplementary Information (Tables S2 and S3).

On analysis with PLS-DA, the best resulting data were achieved by combining data sets using H_3_O^+^, NO^+^ and O_2_^+^ at 12 weeks of age. PLS-DA score plots discriminating between WT littermates and Cushing’s (*het*) mice were determined at (a) 12 weeks of age (no Feature Selection (FS)), data acquired using SIFT-MS, Fig. 3A, and 16 weeks of age including range-scaling (RS1), data acquired by TD-GC-MS, Fig. 3B. According to our findings, the best set of predictors that discriminate between the groups (*het*/ WT) are indicated in Tables 2 and 3 respectively, and these were determined using PLS-DA multivariate analysis.

**Table 2:** PLS-DA statistical analysis in faecal headspace and respective *m*/*z* ions and probable compounds that discriminate the groups (*WT*/*het*).

*m/z* (H_3_O^+^/NO^+^/O_2_^+^)	SIFT-MS
	Compound
47 (H_3_O^+^)	Ethanol/ formic acid
59 (H_3_O^+^)	Acetone/Propanol
65 (H_3_O^+^)	Ethanol
77 (H_3_O^+^)	Acetone
61, 79 (H_3_O^+^)	Acetic acid/propanol
89 (H_3_O^+^)	Butyric acid/acetoin/pyruvic acid/pentanol/methyl propionate
31 (O_2_^+^)	By-product of methanol or ethanol

Data acquired by SIFT-MS (12 weeks). H_3_O^+^/NO^+^/O_2_^+^ denotes the combination of the individual mass spectra pertaining to the three precursor ions into one dataset.

**Table 3:** PLS-DA statistical analysis in faecal headspace and respective retention time (RT) and probable compounds that discriminate the groups (*WT*/*het*). Data acquired by TD-GC-MS (16 weeks). Retention time (RT) is given in minutes and its chemical abstract service number (CAS).

RT (min)	CAS	TD-GC-MS
		Compound
4.277	56893	Unknown
6.585	67641	Acetone
9.707	431038	2,3-Butanedione
10.861	64197	Acetic acid
12.011	105373	Propanoic acid, ethyl ester
12.244	105544	Butyric acid, ethyl ester
15.074	116530	Butyric acid, 2-methyl

SIFT-MS (H_3_O^+^/NO^+^/O_2_^+^) analysis confirmed the presence of significant greater levels of *m/z* 61, 79 (H_3_O^+^) (probably acetic acid since *m/z* 90 (NO^+^) is also present following the feature selection (FS) step) in faecal headspace of Cushing’s (*het*) mice at 12 weeks of age. Remarkably, these findings were supported by the TD-GC-MS analysis where acetic acid was found to discriminate between the groups (*het*/WT) at 16 weeks of age (Table 3). Short-chain fatty acids (SCFAs) (mainly acetate, butyrate and propionate) are produced in the gut by anaerobic microbial fermentation pathways^9^. Acetate is the major end-fermentation product in the human gut formed by acetogenic bacteria^8^. Long-chain alcohols may be originated by reduction of the corresponding acid, therefore acetic acid might be reduced to ethanol in the same manner^31^.

Regarding *m/z* 89 (H_3_O^+^), which is tentatively identified as butyric acid (although it could be other compounds, i.e. acetoin, pyruvic acid, pentanol and methyl propionate)^32^, two different pathways for butyrate production are known in butyrate-producing bacteria in human gut^8,33,34^. The reduced levels of butyric acid (*m/z* 89 (H_3_O^+^)) found in faecal headspace of Cushing’s (*het*) mice might be an indicator of the reduced number of butyrate-producing bacteria in Cushing’s mice gut. There is previous evidence of changes in the gut microbiota of type-2 diabetic patients when compared to non-diabetic subject^17^. An earlier investigation reported that patients with type-2 diabetes had less butyrate-producing bacteria than controls^35,36^. However, ethnic factors, dietary differences, and intake of medication might take an important role on studies’ discrepancies previously reported.

The existence of increased levels of acetone in Cushing’s mice faecal headspace was supported and validated by the TD-GC-MS analysis, and probably arise from-fatty acid and carbohydrate metabolism in the liver. The ion *m/z* 47 (H_3_O^+^) is very likely ethanol, and this results from the breakdown of carbohydrates by bacteria in the gut^9^. Using the chromatographic capabilities of TD-GC-MS, butyric acid, ethyl ester and butyric acid, 2-methyl were identified as predictor ions for the discrimination of the groups (WT/*het*).

There is evidence of three different pathways used by colonic bacteria for propionate formation in human gut, i.e. succinate pathway, acrylate pathway and propanediol pathway. Nevertheless, propionate is mostly formed via the succinate pathway by *Bacteroidetes* and by some *Firmicutes*^8,37^. Significantly increased levels of propanoic acid, ethyl ester were found in Cushing’s (*het*) mice faecal headspace. Faecal SCFAs concentration in healthy humans strongly depend on the dietary regime, medication, age, and genetic background^38^, and elevated levels of SCFAs were observed in obese humans^39^. Other studies have demonstrated that SCFAs directly contribute to host protection from metabolic diseases. In particular, SCFAs were administrated in mice, and the findings suggested that butyrate, propionate and acetate protect against diet induced obesity and insulin resistance^40,41^. Butyrate and propionate, but not acetate, induce gut hormones and reduce food intake, reduces inflammation and consequently insulin resistance^40,42^. Acetic acid seems to beneficially affect glucose metabolism by reducing hyperglycaemia and supressing appetite in mice^43,44^. By contrast, a recent and detailed study reported changes to gut microbiota, and altered faecal SCFAs concentrations which promoted the metabolic syndrome, insulin resistance and obesity, through the activation of the parasympathetic nervous system^14^. Interestingly the authors found increased production of acetate in rodents on a high-fat diet which in turn promoted increased glucose output and stimulated the secretion of insulin and ghrelin hormone triggering increased appetite and promoting obesity. They have also observed plasma and faecal acetate concentrations significantly increased in insulin-resistant rats after three days or four weeks on a high-fat diet, driving obesity and insulin resistance. Our findings are consistent with the previous report^14,15^, wherein differences in acetic acid differ remarkably between the faecal headspace of WT littermates and those of Cushing’s mice, either by SIFT-MS and TD-GC-MS. Cushing’s mice developed obesity and showed significantly higher blood glucose concentrations and plasma insulin concentrations, supporting the hypothesis that Cushing’s mice develop a diabetic phenotype related to a gut microbiota interaction.

#### Discrimination of groups throughout the ageing process – SIFT-MS and GC-MS data

**Figure 4:**
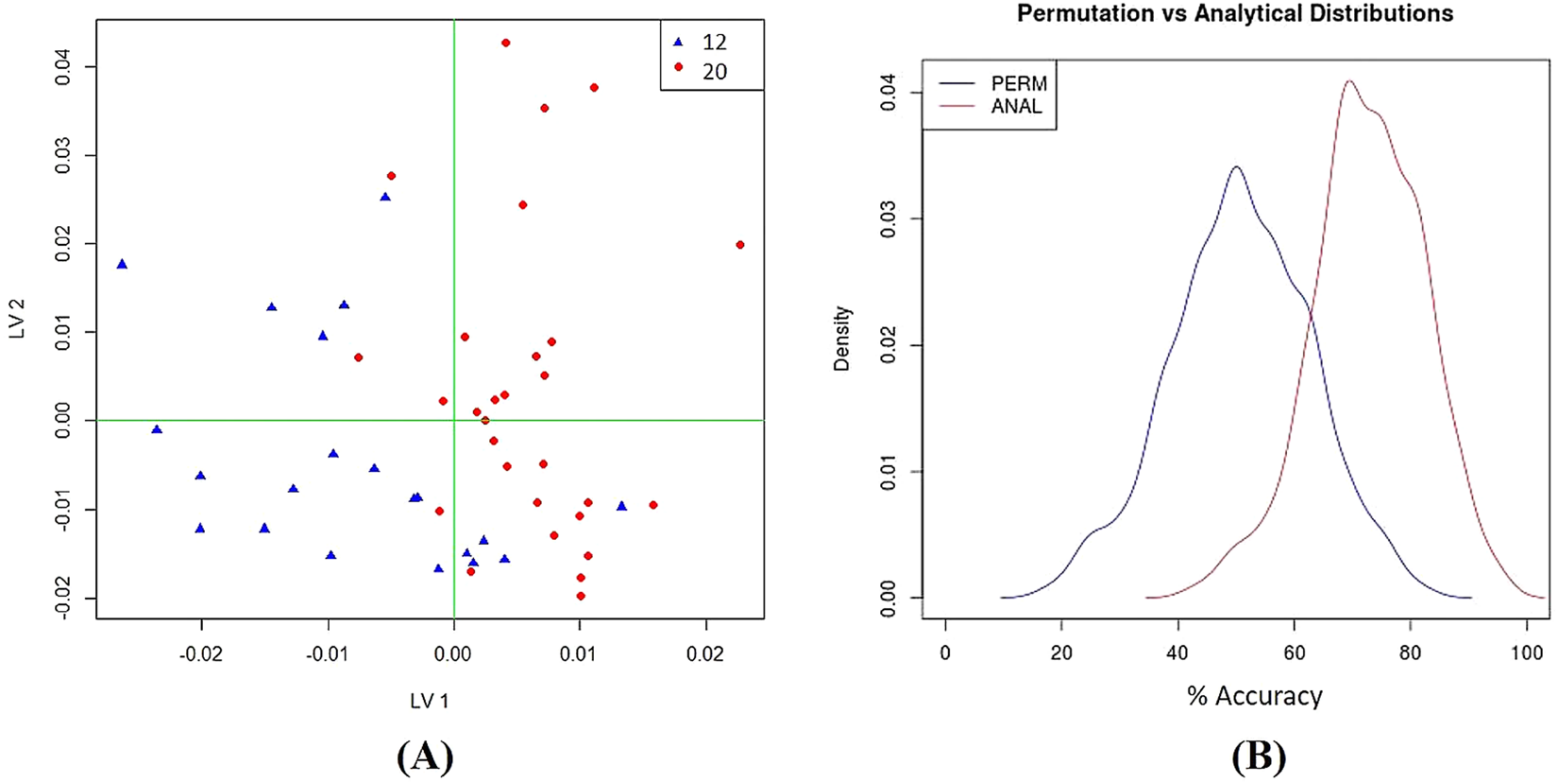
PLS-DA score plot discriminating between 12 and 20 weeks of age in Cushing’s (*het*) mice; highlighted in blue is shown the 12 weeks group and 20 weeks group in red (**A**) Permutation results demonstrating that reported performance metrics were not due to chance (**B**) as illustrated by the distance between the maximum peaks of the two density distributions. Data acquired with SIFT-MS (H_3_O^+^, NO^+^ and O_2_^+^). Number of independent observations and number of variables is given in Supplementary Information (Table S2).

Not only has the discriminant analysis provided insight of the discrimination of groups according to the phenotype, but also whether the volatile profile is able to discriminate groups according to the ageing process. These findings might be linked to previous evidence of age-related changes in gut microbiota composition^45,46^. The best resulting data were achieved by combining data sets using H_3_O^+^, NO^+^ and O_2_^+^ using PLS-DA pre-subjected to principal components analysis (PCA) and discriminating between 12 weeks and 20 weeks of age (Cushing’s (*het*) mice) (Fig. 4A). An overall classification accuracy indicated that 73% grouped cases were correctly classified which had a specificity of 59% and a sensitivity of 83%.

Permutation analysis (Fig. 4B) suggests that the performance metrics obtained were not due to chance. Acetone was found to increase significantly with age. The ions *m/z* 77 (H_3_O^+^) and *m/z* 58 (O_2_^+^) are likely to be acetone and probably arise from-fatty acid and carbohydrate metabolism^31^. The breakdown of carbohydrates by bacteria in the gut is performed by several different genera including *Bacteroides*, *Lactobacillus* and *Bifidobacterium*^47^. The main degradation products are short-chain fatty acids (SCFAs) (mainly acetate, propionate and butyrate), lactate and other products including CO_2_, H_2_, CH_4_ and ethanol^9^. The set of ions responsible for the discrimination include *m/z* 65, *m/z* 83 (H_3_O^+^) and *m/z* 63 (NO^+^), which are likely ethanol, whereas the ions *m/z* 51, *m/z* 69 (H_3_O^+^) are likely methanol. The identity of ion *m/z* 63 (O_2_^+^) has not been established but it was one of the ions responsible for the discrimination.

*Afmid* (*hom*) knockout mice did not present statistically significant differences in their volatile faecal metabolome once compared to their WT littermates, on analysis with PLS-DA. Whereas, discrimination of groups according to the ageing process was observed throughout the age (8–12, 8–16, 12–16 weeks), with classification accuracies exceeding 75%. Based on highest accuracy and highest sensitivity observed between 12 weeks and 16 weeks of age in knockout *Afmid* (*hom*) mice (TD-GC-MS data sets), the best set of compounds that discriminate the two age groups are 2-butanone, 3-hydroxy also known as acetoin; pyridine-3-carboamide and 1,3-dioxalane, 2-methyl. Interestingly, there is evidence that acetoin (2-butanone, 3-hydroxy) is a product of fermentation in bacteria^48,49^. *Bacillus subtilis, Bacillus amyloliquefaciens, Enterobacter cloacae, Serratia marcescens*, and *Paenibacillus polymyxa*, can produce acetoin from pyruvate via α-acetolactate by two enzymatic steps catalysed by α-acetolactate synthase and α-acetolactate decarboxylase^48^, and consequently acetoin can be further converted to 2,3-butanediol^50^. The remaining compounds, pyridine-3-carboamide and 1,3-dioxalane, 2-methyl cannot be easily attributed to any specific metabolic pathways.

#### Comparative analysis of the volatile faecal metabolome in type 2 diabetes mouse models

Our findings suggested that *Afmid* (*hom*) mice and Cushing’s (*het*) mice have differences in regards to their volatile faecal metabolome and the PLS-DA model successfully classified them according to the phenotype. Using SIFT-MS, the loadings responsible in providing the highest discrimination between the groups *Afmid* (*hom*) mice and Cushing’s (*het*) mice are *m/z* 61, *m/z* 79 (H_3_O^+^) tentatively identified as acetic acid, and *m/z* 89 (H_3_O^+^) potentially identified as methyl propionate/ pentanol/ ethyl acetate/ butyric acid. Increased levels of *m/z* 89 (H_3_O^+^) were found in *Afmid* (*hom*) mice once compared to Cushing’s mice, whereas *m/z* 61 and *m/z* 79 increased levels of these ions were found in Cushing’s (*het*) mice, potentially indicating that further decomposition onto butyric acid is limited by the number and survival rate of butyrate-producing bacteria in the mouse gut. Earlier evidence demonstrated the importance of the gut microbiota in the development of obesity and the onset of diabetes in mice, where an increased ratio of *Bacteroidetes* and the *Firmicutes* was found in genetically obese mice and associated with an increased faecal concentration of the major fermentation end-products acetate and butyrate, when compared to lean littermates; and faecal transplants from mice with glucose intolerance into healthy germ-free mice induced glucose intolerance^15,51^.

**Figure 5:**
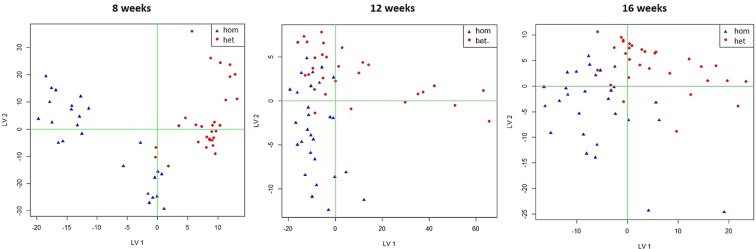
PLS-DA score plot discriminating between *Afmid* (*hom*) mice and Cushing’s (*het*) mice over the age. Data acquired using TD-GC-MS. Highlighted in blue is shown the “*hom*” animals and in red the “*het*” animals. 8 weeks: Classification of 93.1%, specificity 96.5% and sensitivity 90.2%; 12 weeks: Classification of 87.2%, specificity 87.2% and sensitivity 87.2%; 16 weeks: Classification of 91.5%, specificity 92.9% and sensitivity 90.7%. Number of independent observations and number of variables is given in Supplementary Information (Table S3).

The studied mouse models differ in terms of their volatile metabolic profile, and age-related changes in the volatile pattern seem to be significant. PLS-DA score plots showed the discrimination between *Afmid* (*hom*) mice and Cushing’s (*het*) mice over the age (Fig. 5).

Age-related changes between *Afmid* (*hom*) mice and Cushing’s (*het*) mice were investigated here and overall good classification results ranging from 93.1% (8 weeks), specificity 96.5% and sensitivity 90.2%; 87.2% (12 weeks) specificity 87.2% and sensitivity 87.2% and 91.5% (16 weeks) specificity 92.9% and sensitivity 90.7% were determined. The volatile metabolic profile of the animals seem to differ the most in an early life stage (8 weeks) further developing diverse volatile patterns over the age (up to 16 weeks of age). The set of compounds responsible for discriminating longitudinal changes in the volatile faecal metabolome between the two different mouse models is indicated in Table 4. These are the most likely identification of compounds, however the match is low in some cases and the metabolic pathways for some are unclear.

**Table 4:** Set of predictors discriminating longitudinal changes in the volatile faecal metabolome between *Afmid* (*hom*) mice and Cushing’s (*het*) mice.

Age (weeks)	RT (min)	Most probable compound	AMDIS (%)
8	3.093	Unknown	14.1
	3.950	Unknown	38.9
	4.145	Acetaldehyde	75.5
	10.053	2,3-butanedione	74.9
	23.877	2-hexadecanol	33.5
	25.147	Bicyclopropyl-2-octanoic acid	15.3
12	4.308	Unknown	32.8
	6.806	Acetone	88.4
	9.545	2,3-butanedione	64.3
	5.783	Pentane	13.3
	17.804	Piperidine, 1,3-dimethyl	37.5
	19.383	Cyclopropanetetradecanoic acid	22.3
	19.906	Benzene, 1,3-bis(1,1-dimethylethyl)-	12.3
16	4.220	Acetaldehyde	25.0
	9.459	2,3-butanedione	78.6
	10.513	Acetic acid, ethanyl ester	15.5
	17.957	Nonanal	35.4
	15.185	Unknown	8.64

TD-GC-MS data analysed using PLS-DA. Retention time (RT) is given in minutes, AMDIS probabilities (%).

## Conclusion

The present study aimed to investigate the volatile faecal profile in two type 2 diabetes mouse models – Cushing’s syndrome and single *Afmid* knockout mice – using selected ion flow tube mass spectrometry (SIFT-MS) and gas chromatography-mass spectrometry combined with thermal desorption (TD-GC-MS).

Univariate and multivariate data analysis, complementary statistical techniques, have proven to be a robust tool in the study of gut metabolomics. The gut microbiota of Cushing’s diabetic mice seem to have a different composition once compared to wild-type (WT) littermates, and statistically significant differences in volatile faecal metabolites, including short-chain fatty acids (SCFAs), alcohols and ketones. *Afmid* (*hom*) knockout mice did not present statistically significant differences in their volatile faecal metabolome when compared to their respective WT littermates.

Discriminant analysis upon the ageing group demonstrated the incidence of changes in the longitudinal volatile faecal metabolome of Cushing’s diabetic mice, mainly attributed to methanol, ethanol and acetone. Longitudinal volatile changes in *Afmid* mice were shown to be attributed to 2-butanone, 3-hydroxy also known as acetoin; pyridine-3-carboamide and 1,3-dioxalane, 2-methyl.

A comparative multivariate data analysis of the volatile faecal metabolome in type 2 diabetes mouse models showed differences mainly in terms of acetic acid and butyric acid. Reduced levels of *m/z* 89 (H_3_O^+^) tentatively identified as butyric acid were found in Cushing’s mice once compared to both WT littermates and *Afmid* mice, potentially indicating that further decomposition onto butyric acid is limited by the number of butyrate-producing bacteria in the animals’ gut. While, *m/z* 61 and *m/z* 79 (H_3_O^+^) (tentatively identified as acetic acid) increased levels of these ions were found in diabetic Cushing’s mice faecal headspace once compared to both WT littermates and *Afmid* mice, which is consistent with earlier reported. All the animals were housed in the same environment and fed *ad libitum* on the same commercial diet, and importantly, WT littermates present an identical genetic background as to the respective diabetic animals. Therefore, these findings suggest that mice developed a diabetic phenotype and that the altered VOC profile may imply a related change in gut microbiota, particularly in Cushing’s mice. Furthermore, this study provided major evidence of age-related changes on the volatile profile of diabetic mice.

## Methods

### Animals

Male heterozygous (*het*) Crh^−120/+^ mice on a B6-C3PDE F1 background were backcrossed to B6-C3PDE F1 female wild-type mice to produce progeny which were screened for phenotypes and classified as mutant Cushing’s (*het*) mice and wild-type (WT) littermates. A cohort of 65 animals (21 male and 44 female) was used of whom 30 were genotyped as mutants (*het*) and 35 WT littermates^6^.

A cohort of 48 homozygous (*hom*) knockout (*Afmid*^tm1b^/^tm1b^) mice (24 male and 24 female) on a C57BL6/NTac background were bred which were screened for phenotypes of whom 30 were genotyped as mutants (*hom*) and 18 wild-type (WT) littermates.

Mice were maintained in controlled light (12 h light and dark cycle), temperature (21 ± 2 °C) and humidity (55 ± 10%). Mice had free access to water (10–12 ppm chlorine) and were fed *ad libitum* on a commercial diet (5.3% fat [corn oil], 21.2% protein, 57.4% carbohydrate, 4.6% fibre; Rat and Mouse Diet No. 3 (RM3), Special Diet Services, Essex, UK)^6^. All mice were housed in groups of 2–5 mice of the same gender. Adult (8 weeks of age) Cushing’s mice were tested over four months (8, 12, 16 and 20 weeks of age) while adult (8 weeks of age) *Afmid* mice were tested over a three month period (8, 12 and 16 weeks of age). Stool pellets were collected from all the animals and the VOCs emanated from the stool pellets were analysed further. Blood glucose analysis was carried out simultaneously (details are given below).

All animal studies were carried out using guidelines issued by the United Kingdom (UK) Medical Research Council (MRC), in Responsibility in use of Animals for Medical Research (July 1993), and the requirements of Home Office Project License number 30/3146. The experimental protocols were approved by the MRC Harwell Institute Animal Welfare and Ethical Review Body (AWERB). All animal studies were carried out at the Mary Lyon Centre (Harwell, UK)^6^.

### Stool collection

Stool samples were collected from each mouse at various times for analysis of VOCs. Animals were individually housed in metabolic cages (Techniplast Kettering, UK) for 30 minutes or until sufficient gathering of stool, i.e. six pellets per animal. The mice were fed *ad libitum* on water (10–12 ppm chlorine) and powdered chow (RM3, Special Diet Services Essex, UK). Autoclavable red transparent igloos (Datesand Ltd, Manchester, UK) were provided as environmental enrichment. Faecal samples were collected into Eppendorf containers, and these were immediately frozen at −20 °C. Faecal samples were then transported to The Open University in dry ice and kept at −80 °C until further analysis.

### Blood and plasma biochemistry

Animal bodyweight was measured (precision balance OHAUS Explorer Pro EP213 ± 0.001 g accuracy) and recorded prior to analysis and over the duration of the study. Blood samples were taken on the same days as faecal samples and were taken from the lateral tail vein following application of topical local anaesthesia (EMLA cream) leaving it to work for 15 minutes, and while mice were restrained in a Perspex bleeding tube. Blood glucose levels were measured at the same period of the day, specifically between 2 pm and 4 pm, using a blood glucose monitoring system (AlphaTRAK2)^6^.

In order to evaluate the insulin levels in mice, plasma concentrations in blood were measured after terminal bleeding of the animals. Therefore, the plasma insulin concentrations for Cushing’s mice and respective WT littermates do not correspond to 20 weeks of age but a later stage once the animals were culled, i.e. animals born in April and May were culled at 28 weeks of age, and animals born in June were culled at 24 weeks of age. Similarly, plasma insulin concentrations for *Afmid* mice and respective WT littermates do not correspond to 16 weeks of age, i.e. animals born in March were culled at 32 weeks of age, and animals born in May were culled at 28 weeks of age. An enzyme-linked immunosorbent assay (ELISA) kit (Cat no. EZRMI-13K) was used and the range of the assay set between 0 and 15 ng mL^−1^. The insulin concentration of three animals were above the upper limit for the ELISA assay thus a value of 15 ng mL^−1^ was assigned for these animals^6^.

### VOC sampling and faecal headspace analysis

Selected ion flow tube mass spectrometry (SIFT-MS) enables direct, real-time monitoring of VOCs, and the SIFT analytical technique has been described in detail previously^52^. Data were collected using the Mk2 instrument (PDZ Europa, UK) with a flow rate of corresponding to a pressure of 0.008 Torr (the flow tube pressure of 1 mbar (0.7 Torr) and a flow rate of 53 sccm). Six pellets per animal were placed inside Nalophan bags (35 cm long Nalophan sampling bag, made up of 65 mm diameter Nalophan NA tubing 25 µm thick (Kalle UK)); sample bags were sealed and filled with hydrocarbon free air (Air Products); after 1 hour of incubation at 37 °C, the bags were connected to the SIFT-MS via the heated sampling line and the faecal headspace further analysed. The normalisation of the data corrects for the variation in sample weight. Samples were analysed in random order and full spectra of the count rates in the range *m/z* 10 to *m/z* 140 were recorded for all the samples via H_3_O^+^, NO^+^ and O_2_^+^ precursor ions, using the Full Scan mode and a total sample time of 30 seconds. Background laboratory air was also analysed.

After the faecal headspace analysis by SIFT-MS, the sample headspaces containing VOCs were pumped into pre-conditioned stainless-steel thermal desorption sorbent tubes (Markes International Ltd) and analysed by gas chromatography-mass spectrometry (GC-MS) coupled to thermal desorption (TD). The detailed approach is given below.

Gas chromatography-mass spectrometry combined with thermal desorption (TD-GC-MS) has been widely used for trace gas analysis in human health monitoring^24,53–55^ and the working principle has been described in detail elsewhere^56^. VOCs were pumped into pre-conditioned stainless-steel TD sorbent tubes for 5 min at a constant flow of 100 mL min^−1^ (Pump TSI Inc. SidePak Model SP730). These stainless-steel tubes without inert coating were used and had dual packing comprising of 40% Tenax TA and 60% Carbotrap (Markes International Ltd). Prior to analysis, the tubes were spiked with 1 µL of internal standard, d8-toluene (50 ng) in methanol, and were then flushed with nitrogen for 30 sec. All samples were analysed in random order according to the method described below.

Chromatographic analyses were performed using an Agilent 6890/5973 GC-MS system equipped with an Ultra 2 autosampler and UNITY model 1 thermal desorber (Markes International Ltd). The tubes were submitted to a pre-purge of 1.0 min, followed by desorption at 260 °C for 3.0 min. The trap temperature was set at −7 °C and the actual trap desorption occurred at 300 °C for 3.0 min. The volatiles were separated using an Rxi-624 Sil MS column (60 m × 0.32 mm, film thickness 1.8 µm, Restek) working in a constant flow mode. The column temperature program involved an initial increase from 35 °C to 60 °C at a rate of 11 °C min^−1^, followed by a rate of 20 °C min^−1^ up to 220 °C, and a constant temperature of 220 °C for 10 min. The mass spectrometer was operated in a SCAN mode with an associated *m/z* range set from 33.0 to 260.0. The transfer line, ion source and quadrupole temperature were kept at 230 °C, 230 °C and 150 °C, respectively.

### Data processing

Prior to data analysis, the counts per second acquired by SIFT-MS were normalised against the counts per second of the H_3_O^+^ (*m/z* 19) precursor ions. The H_3_O^+^ (*m/z* 19) ions react with the gas sample producing the hydrated ions H_3_^16^O^+^(H_2_O)_n_, n = 1,2,3 corresponding to *m/z* 37, 55, 73. The humidity levels found in the gas samples were evaluated, i.e. the average fraction of counts per second between the ions *m/z* 19, 37, 55, 73 and respective isotopes, ^17^O (*m/z* 20, 38, 56, 74), and ^18^O (*m/z* 21, 39, 57, 75) according to earlier literature^57^. The fractions of the larger cluster ions will likely increase with increasing water vapour^57^. The fractions remained relatively constant (same order of magnitude) across the different age groups (Tables S4 and S5 given in Supplementary Information). This provides reasonable evidence that humidity does not vary too much in the samples, therefore the authors consider this is not critical, although normalisation to the total ion count would be more accurate.

All *m/z* values pertaining to the known isotopologue and hydrate ions (i.e. *m/z* values: 19, 21, 30, 32, 34, 37, 39, 48, 55, 57, 66, 73, 75, and 91) were removed, except for the linear discriminant analysis (LDA)/canonical discriminant functions. Principal components analysis (PCA)^58^ was employed to visually identify any outlying samples, and to carry out feature selection (FS) by omitting redundant variables. For comparison, no feature selection was also carried out. There was little improvement in specificity and sensitivity when using the feature selection.

TD-GC-MS data analysis was performed through the aid of AMDIS (Automated Mass Spectral Deconvolution and Identification System) software, and followed by reliable identification of compounds using the NIST (National Institute of Standards and Technology) library. Prior to multivariate statistical data analysis, the data was normalised against the internal standard. Principal components analysis (PCA)^58^ was employed to visually identify any outlying samples. The chromatographic peaks were aligned via the correlation optimised warping (COW) algorithm^59,60^.

For both SIFT-MS and GC-MS, additional scaling was carried out to investigate the effects on the performance metrics; these involved auto-scaling (AS), range-scaling (RS) and normalisation (NRM)^61^.

### Statistical analysis

Univariate statistical analysis was carried out using student’s t-test (normally distributed data); Mann-Whitney U test, Wilcoxon Signed Rank Test using a Bonferroni adjusted p value (p < 0.001) and Friedman test were used when appropriate for “distribution-free” data; and one-way repeated measures ANOVA with Bonferroni post-hoc test. Testing corrections such as Bonferroni post-hoc tests are used to reduce the number of false positives.

Multivariate statistics was performed and a predictive model was built for group membership by linear discriminant analysis (LDA)^62^, such model is based on combinations of the predictor variables that provide the best discrimination between the groups. Follow-up using canonical discriminant functions was performed. All the variables (*m/z*) were transformed using the square root transformation to convert the distribution from “skewed” to “normal”, followed by standardization of the data sets, i.e. *m/z* values were mean-centred and divided by the respective standard deviation of each data set. The univariate statistical analysis, LDA and canonical discriminant functions were performed using the software IBM SPSS Statistics 21.0.

LDA was performed, where two or more groups are known *a priori* and these are classified into one of the known populations based on the measured features. LDA is a supervised technique in which a mathematical model is created in which patterns within the independent data (e.g. SIFT-MS *m/z* ions) are related to the dependent (response) variables, in this case the population groups consisting of wild-type and either *“het”* for Cushing or “*hom”* for *Afmid*. LDA is also said to be a “hard classifier” in which there needs to be a distinctive (linear) separation between the two population groups.

Partial least squares-discriminant analysis (PLS-DA)^63^ works in the same manner as LDA but is said to be a “soft classifier” in which samples are classified into the population groups by probability. The partial least squares (PLS) decomposes the independent data into a scores and loadings matrix in a similar approach to principal components analysis (PCA)^58^, but in which latent variables (LVs) form a new coordinate system in which the first latent variable (i.e. LV1) contains the most characteristic information pertaining to the data, i.e. has the highest variance^63^. The PLS scores relate the population groups whilst the PLS loadings indicate the significant variables, e.g. SIFT-MS *m/z* ions, that contribute to the distinction between the two population groups.

PLS-DA was performed in conjunction with bootstrapping. This involved randomly splitting the dataset into two groups: 70% as a training set; 30% as a testing set, ensuring both sets were balanced. An optimum PLS-DA model was attained via optimisation with leave-one-out cross-validation (LOO-CV). This optimum model is then used to predict the classification of the testing set. The performance metrics of the model is determined by calculating the accuracy, specificity and sensitivity. The process is repeated 150 times to attain an overall performance. A script written in R software^64^ carried out the analyses using the pls.lda() function from the *plsgenomics* package^65^. At the same time, permutation analysis was performed in which the population groups were randomised in order to ensure that the performance metrics attained above were not due to chance.

### Ethical approval

All applicable international, national, and/or institutional guidelines for the care and use of animals were followed. All procedures performed in studies involving animals were in accordance with the ethical standards of the institution or practice at which the studies were conducted.

## Supplementary information

Supplementary Information

## Data Availability

The datasets generated during and/or analysed during the current study are available from the corresponding author on reasonable request.
