# Supplementary material for: Monitoring type 2 diabetes from volatile faecal metabolome in Cushing’s syndrome and single Afmid mouse models via a longitudinal study
Source: Sci Rep. 2019 Dec 11;9:18779. doi: 10.1038/s41598-019-55339-9 (PMC6906526; doi:10.1038/s41598-019-55339-9)
Supplement: Supplementary file 1 — Supplementary Information [file 41598_2019_55339_MOESM1_ESM.doc]

**Monitoring type 2 diabetes from volatile faecal metabolome in Cushing´s syndrome and single *Afmid* mouse models via a longitudinal study**

**Célia Lourenço^1, 2^, Darren Kelly^3^, Jack Cantillon^3^, Michael Cauchi^3^,** **Marianne A. Yon^4^, Liz Bentley^4^, Roger D. Cox^4^ and Claire Turner^1, 5*^**

1 School of Life, Health & Chemical Sciences, Faculty of Science, Technology, Engineering and Mathematics, The Open University, Walton Hall, Milton Keynes, MK7 6AA, UK.

2 Now at: School of Water, Energy and Environment, Cranfield University, Cranfield, Bedfordshire MK43 0AL, UK.

3 Department of Mathematics & Statistics, University of Limerick, Limerick, V94 T9PX, Ireland.

4 Mammalian Genetics Unit, MRC Harwell Institute, Harwell Campus, Oxfordshire, OX11 0RD, UK.

5 Now at: College of Health & Life Sciences, Brunel University London, Kingston Lane, Uxbridge, Middlesex, UB8 3PH, UK.

*Corresponding author: E-mail address: claire.turner@brunel.ac.uk ORCID ID: https://orcid.org/0000-0003-3613-545

**Supplementary Information**

**Table S1.** Number of independent observations and number of variables included in the statistical analysis of SIFT-MS datasets for the determination of LDA/canonical discriminant functions.

| **Dataset SIFT-MS** |  | **Independent Observations** | | | **Variables ^a^** |
| --- | --- | --- | --- | --- | --- |
| **Ion** | **Weeks** | ***WT*** | ***het*** | ***hom*** | ***m/z*** |
| **H_3_O^+^** | 8 | 53 | 30 | 30 | 123 |
|  | 12 | 53 | 30 | 30 |  |
|  | 16 | 53 | 30 | 30 |  |
|  | 20 | 35 | 30 | N/A |  |
| **NO^+^** | 8 | 53 | 30 | 30 | 116 |
|  | 12 | 41 | 24 | 30 |  |
|  | 16 | 53 | 30 | 30 |  |
|  | 20 | 35 | 30 | N/A |  |
| **O_2_^+^** | 8 | 53 | 30 | 30 | 116 |
|  | 12 | 41 | 24 | 30 |  |
|  | 16 | 52 | 30 | 30 |  |
|  | 20 | 35 | 30 | N/A |  |

^a^ Isotopologue and hydrate ions (i.e. *m/z* values: 19, 21, 30, 32, 34, 37, 39, 48, 55, 57, 66, 73, 75, and 91) were not removed. Variables equal to zero were removed from the dataset. Key: N/A – non-applicable.

***Table S2. Number of independent observations and number of variables included in the statistical analysis (PLSDA) of SIFT-MS datasets.***

| **Dataset** | **Independent Observations** | | **Variables** |
| --- | --- | --- | --- |
| **SIFT-MS** | **Group 1** | **Group 2** | ***m/z*** |
| Cushing (Het): 12 weeks (PPCA*) | WT: 21 | HET: 21 | 293 |
| Cushing (Het): 12 weeks (PPCA_FS**) | WT: 21 | HET: 21 | 59 |
| Cushing (Het): Longitudinal (PPCA: 12 v 20 weeks) | 12w: 21 | 20w: 29 | 298 |
| Cushing (Het): Longitudinal (PPCA_FS: 12 v 20 weeks) | 12w: 21 | 20w: 29 | 59 |

Key: *PPCA = Post-PCA for removal of outliers; FS** = Feature selection that follows PPCA for removal of redundant variables.

Feature Selection using PCA for Removal of Insignificant Variables:

Following removal of any outliers via principal components analysis (PCA), leading to a new dataset (PPCA), feature selection was carried out by using PCA again to decompose the PPCA dataset into a scores and loadings matrix. The PC1 loadings were extracted from the loadings matrix (namely the first row of the loadings matrix) as this corresponds to the highest variance captured. The PC1 loadings should therefore contain the key variables that are highly influential on the groupings of the observations (contained in the scores matrix). In order to select only the key variables, a threshold is established akin to setting a significance level in hypothesis testing. In this work, the threshold was set at 10%. This means that any variables whose values were above this threshold were deemed to be significant, and the corresponding columns (i.e. features) were extracted from the PPCA dataset to form a new dataset (e.g. PPCA_FS where FS denotes feature selection) with fewer columns than the PPCA dataset.

**Table S3.** Number of independent observations and number of variables included in the statistical analysis (PLSDA) of GC-MS datasets.

| **Dataset**  **GC-MS ^a^** | **Observations** | | **Variables** |
| --- | --- | --- | --- |
|  | **Group 1** | **Group 2** | **Retention Time** |
| Cushing (Het): 12 weeks (PPCA) | WT: 34 | HET: 28 | 14,114 |
| Cushing (Het): 16 weeks (PPCA) | WT: 35 | HET: 27 | 14,114 |
| Afmid (HOM) v Cushing (HET): 8 Weeks | HOM: 25 | HET: 30 | 14,114 |
| Afmid (HOM) v Cushing (HET): 12 Weeks | HOM: 28 | HET: 29 | 14,114 |
| Afmid (HOM) v Cushing (HET): 16 Weeks | HOM: 28 | HET: 30 | 14,114 |

^a^ No feature selection was performed on the GC-MS data.

| **Table S4.** Averaged fraction of counts/s for the ions *m/z* 19, 37, 55, 73 and respective isotopes, ^17^O (*m/z* 20, 38, 56, 74), and ^18^O (*m/z* 21, 39, 57, 75). Data acquired for *het*/*WT* groups across the age period. | | | | | | |
| --- | --- | --- | --- | --- | --- | --- |
| **Age (weeks)** | ***m/z* [20]/[19]** | ***m/z* [38]/[37]** | ***m/z* [56]/[55]** | ***m/z* [57]/[55]** | ***m/z* [74]/[73]** | ***m/z* [75]/[73]** |
| 8 | 0.0011 | 0.0013 | 0.0021 | 0.0080 | 0.0044 | 0.0218 |
| 12 | 0.0010 | 0.0014 | 0.0022 | 0.0213 | 0.0085 | 0.1315 |
| 16 | 0.0012 | 0.0016 | 0.0017 | 0.0134 | 0.0059 | 0.0443 |
| 20 | 0.0013 | 0.0013 | 0.0035 | 0.0109 | 0.0025 | 0.0179 |

| **Table S5.** Averaged fraction of counts/s for the ions *m/z* 19, 37, 55, 73 and respective isotopes, ^17^O (*m/z* 20, 38, 56, 74), and ^18^O (*m/z* 21, 39, 57, 75). Data acquired for *hom*/*WT* groups across the age period. | | | | | | |
| --- | --- | --- | --- | --- | --- | --- |
| **Age (weeks)** | ***m/z* [20]/[19]** | ***m/z* [38]/[37]** | ***m/z* [56]/[55]** | ***m/z* [57]/[55]** | ***m/z* [74]/[73]** | ***m/z* [75]/[73]** |
| 8 | 0.0010 | 0.0017 | 0.0034 | 0.0121 | 0.0048 | 0.0221 |
| 12 | 0.0022 | 0.0018 | 0.0290 | 0.0113 | 0.1369 | 0.0083 |
| 16 | 0 | 0.0044 | 0.0401 | 0.0802 | 0.2724 | 0.1302 |
